# A Comprehensive Online Calculator for Pediatric Endocrinologists: ÇEDD Çözüm/TPEDS Metrics

**DOI:** 10.4274/jcrpe.4526

**Published:** 2017-06-01

**Authors:** Korcan Demir, Samim Özen, Ergun Konakçı, Murat Aydın, Feyza Darendeliler

**Affiliations:** 1 Dokuz Eylül University Faculty of Medicine, Division of Pediatric Endocrinology, İzmir, Turkey; 2 Ege University Faculty of Medicine, Division of Pediatric Endocrinology, İzmir, Turkey; 3 Ege University Faculty of Medicine, Department of Biostatistics and Medical Informatics, İzmir, Turkey; 4 Ondokuz Mayıs University Faculty of Medicine, Division of Pediatric Endocrinology, Samsun, Turkey; 5 İstanbul University İstanbul Faculty of Medicine, Division of Growth, Development, and Pediatric Endocrinology, İstanbul, Turkey

## To The Editor,

Pediatricians need to make many medical calculations during follow-up of both healthy and sick children. This is particularly relevant for pediatric endocrinologists. The World Health Organization (WHO) provides different tools for offline use in order to assess various anthropometric variables (1). Calculators for many specialties are available in Up To Date which requires subscription (2). There have also been a number of promising and inspiring attempts in our country to make such calculations using Excel or other programming language, however, they could not be widely used. We have recently launched an online, freely accessible, computerized, user-friendly, and scientific tool set containing a wide array of formulae in order to meet the needs of pediatric endocrinologists: ÇEDD ÇÖZÜM (www.ceddcozum.com). ÇEDD stands for Çocuk Endokrinolojisi ve Diyabet Derneği, the official society of pediatric endocrinologists in Turkey and ÇÖZÜM means solution. TPEDS stands for Turkish Pediatric Endocrinology and Diabetes Society and METRICS denotes the nature of the online tool. For international use, we chose a simpler and memorable name for the website: www.childmetrics.org.

ÇEDD ÇÖZÜM (TPEDS METRICS) mainly provides assessment of various physical growth variables. On the main page (Auxology), standard deviation (SD) scores and percentile values can be calculated for weight, height, body mass index, and head circumference of children using one of the three reference data: Centers for Disease Control (CDC), Neyzi et al, and the WHO (weight data are present only for children <10 years of age) (3,4,5,6). Body surface area is calculated according to Costeff method (7). For target height, firstly, mean of paternal and maternal height was calculated; next, 6.5 cm was added to and subtracted from that mean value for boys and girls, respectively (8). Estimation of target height SD scores is done by analyzing the calculated target height using the data of the oldest age group available in the selected reference data (WHO: 18 years, Neyzi et al: 19 years, and CDC: 20 years) (3,4,5,6). At the bottom of the results, there are four links to related pages.

- Growth charts: The input can be seen on the relevant charts derived from the selected reference data.

- Results can be seen in text format.

- Further anthropometric assessments: SD scores of upper/lower segment ratio (for girls between 3-18 years of age and for boys between 2-18 years of age), waist circumference (<17 years of age), and sitting height/height ratio (6-17 years of age) are calculated according to Turkish references (9,10,11,12).

- Calculation of predicted adult height can be made using Bayley-Pinneau (for children with ≥6 years of bone age but the extent of difference between chronological and bone age might prevent calculation for some age groups, especially in boys) and Roche-Wainer-Thissen methods (from 1 year of chronological age to 16 years for boys and 14 years for girls) (13,14).

Growth velocity rates and SD scores can be calculated on another page using four reference data including Turkish (unpublished Neyzi et al data for boys between 10 and 15 years of age and girls between 8 and 13.5 years of age), American [Baumgartner et al (15): 6-month to 15.5-year-old boys and 6-month to 13.5-year-old girls; Kelly et al (16): 5.5-year to 18.5-year-old boys and 5.5-year to 17.5-year-old girls] and WHO data (for children <24 months) (17). Growth section also includes a page for calculation of SD scores of insulin-like growth factor 1 (IGF-1) and IGF-binding protein-3 levels (generated with chemiluminescence method) using the data from 1-17-year-old healthy Turkish children (18). A tool to estimate growth hormone dose is also available.

Bone section provides an opportunity to calculate SD scores for total L1-L4 areal bone mineral density using the data obtained with dual X-ray absorptiometry from healthy Turkish children between 2 and 18 years of age (19). Tubular excretion of phosphate and calcium can be estimated as well.

Calculations of thyroid volume SD score are made according the two Turkish studies. Data from newborns and older children up to 19 years of age are derived from the reports by Mutlu et al (20) and Aydıner et al (21), respectively. Ovarian volume is estimated according to the following formula: x*y*z*0.523 (22,23).

Glucose/insulin ratio, homeostatic model assessment for insulin resistance, and Quick index can be calculated on the Obesity section. Testosterone/dihydrotestosterone and testosterone/androstenedione ratios can be computed on the human chorionic gonadotropin test section. Unit converter is a simple tool for commonly used laboratory variables (24).

SD scores for a given measurement (x) are mainly calculated using LMS data with following formulae: L≠0, SD score =[(x/M)**L–1]/LS or L=0, SD score=ln(x/M)/S (3,4,5,6,10,11,12,16,17,25). Interpolation by weighted mean is used to obtain L, M, and S values at finer intervals that are not provided in the relevant references (26). When no LMS data are present for a variable, SD scores for a given measurement (x) are obtained by the following formula: SD score= (x–mean)/SD (9,15,18,19,20,21). Percentile values corresponding to calculated SD scores are obtained from a standard normal distribution table.

The tool is under protection of our national society, will be kept updated, and will incorporate new features.
